# Undifferentiated embryonal sarcoma of the liver in a child: A case report and review of the literature

**DOI:** 10.3892/ol.2012.1087

**Published:** 2012-12-19

**Authors:** JIE GAO, LIMING FEI, SHENG LI, KAI CUI, JIANBO ZHANG, FACHANG YU, BO ZHANG

**Affiliations:** 1 Department of Hepatobiliary Surgery, Shandong Cancer Hospital, Jinan 250117;; 2 Department of Hepatobiliary Surgery, Yantaishan Hospital, Yantai 264025;; 3 Key Laboratory for Rare and Uncommon Diseases of Shandong, Shandong 250062;; 4 Department of Pathology, Shandong Cancer Hospital, Jinan 250117;; 5 Shandong Academy of Medical Sciences, Shandong 250031, P.R. China

**Keywords:** liver, undifferentiated embryonal sarcoma of the liver, liver tumor

## Abstract

Over 200 cases of undifferentiated embryonal sarcoma of the liver (UESL) have been reported since 1978 when this disease was first described. In the present study, we describe a case of UESL in a 7-year-old female, whose initial symptoms included swelling in the upper abdomen and a palpable enormous irregular tumor. A magnetic resonance imaging (MRI) examination revealed a massive focal lesion in the right lobe of the liver. Hepatic malignant tumor with a high possibility of hepatoblastoma was diagnosed. The tumor was surgically removed and confirmed to be UESL by postoperative pathology and immunohistochemical staining analysis. The patient then received chemotherapy consisting of three cycles of epirubicin (20 mg, days 1–2) and cisplatin (15 mg, days 1–3). To date, the patient has survived for 22 months, and is currently in a good general condition without evidence of local metastasis or recurrence. Although UESL has a high malignancy and a poor prognosis, cases of long-term survival with improved diagnosis and therapy have recently been reported. Therefore, it has been proposed that UESL should not be considered as an hepatic tumor with a poor prognosis. Total resection with preoperative or postoperative radio-chemotherapy is currently considered to be the key approach to improving the survival rate.

## Introduction

Undifferentiated embryonal sarcoma of the liver (UESL), also known as malignant mesenchymoma or embryonic sarcoma, is a rare disease (1) that rarely occurs in adults; 90% of UESL cases occur in children aged 6–10 years (2). There is no evident gender discrepancy in the disease. UESL is often misdiagnosed as other types of hepatic malignant tumors. The diagnosis of UESL relies on postoperative pathology and immunostaining analysis (3). Due to its high malignancy, UESL often metastasizes to the lung, peritoneum and pleura, resulting in a poor prognosis (4). Currently, therapy for UESL mainly comprises surgery with postoperative radio-chemotherapy (5). In the current study, we present a case of UESL. The patient’s family provided consent for the study.

## Case report

A 7-year-old female child was admitted to the Department of Hepatobiliary Surgery of the Shandong Cancer Hospital (Jinan, China) on December 29 2009, with an augmented swelling in the upper abdomen and a palpable massive irregular tumor that had been identified >10 days previously. A magnetic resonance imaging (MRI) scan revealed a massive focal lesion in the right lobe of the liver. The lesion was diagnosed as a malignant tumor with a high possibility of hepatoblastoma. The patient did not present with fever, bellyache, abdominal distention, diarrhea, jaundice, nausea or vomiting.

Physical examination revealed that scleral or systematic mucocutaneous icterus and swollen lymph nodes were not present. The patient had ectasia of the upper abdomen, particularly in the right superior belly. The liver under the rib edge was palpable for 7 cm, and the surface tuberculum crossing the left of the ventral median line was not palpable for 1 cm. No percussion pain was observed in the hepatic renal region.

The laboratory examination results were as follows: α-fetoprotein (AFP), 0.853 ng/ml; carbohydrate-associated antigen (CA) 19-9, 23.39 U/ml; carcinoembryonic antigen (CEA), 0.788 ng/ml; alanine transaminase (ALT), 68.3 *μ*mol/l; aspartate transaminase (AST), 63.8 *μ*mol/l; albumin, 32.01 g/l; albumin and globulin ratio, 1.25; total bilirubin (Tbil), 19.7 *μ*mol/l; direct bilirubin (Dbil), 2.5 *μ*mol/l; indirect bilirubin (Ibil), 17.2 *μ*mol/l; alkaline phosphatase (ALP), 162 *μ*mol/l; γ-glutamyl transpeptidase (GGT), 80 *μ*mol/l.

**Figure 1:**
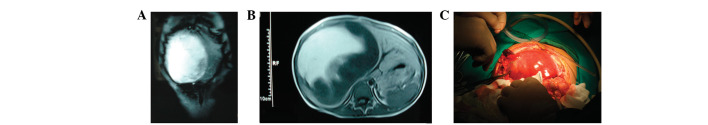
Abdominal magnetic resonance imaging (MRI) examination. (A and B) MRI examination revealed a massive tumor (15×13×10 cm) in the right lobe of the liver, which showed a high central and low peripheral mixed signal shadow on the T1-weighted image (T1W1) and a mixed high signal on T2W1. Certain partitions were observed to be present in the interior of the tumor, and the boundaries remained clear. The patient’s right kidney was pushed down by the pressure, which enhanced the scanning of the peripheral solid parts with a strap type of persistent potentiation; the boundaries were clear and no abnormalities were evident in the left lobe. (C) A small level of clear and transparent pale yellow ascites at a volume of 80 ml was present in the abdominal cavity. A massive tumor with a smooth and glossy surface was evident in the right hepatic lobe, and the Glisson’s capsule remained intact. Moreover, in the patient’s left hepatic lobe, the first and second porta hepatis have been removed by the pressure, and the inferior vena cava has been distorted.

The MRI examination revealed a massive tumor (15×13×10 cm) in the right lobe of the liver (Fig. 1A and B), which demonstrated a high central and low periphery mixed signal shadow on the T1-weighted image (T1W1) and a mixed high signal on T2W1. Certain partitions were observed to be present in the interior of the tumor and the boundaries remained clear. The patient’s right kidney was pushed down by the pressure, which enhanced the scanning of the peripheral solid parts with a strap type of persistent potentiation; the boundaries were clear and no marked abnormalities were observed in the left hepatic lobe. Following admission, the patient underwent partial excision of the right hepatic lobe and the left inner lobe on December 2, 2009. The perioperative results revealed that a small level of clear and transparent pale yellow ascites at a volume of 80 ml was present in the abdominal cavity (Fig. 1C). A massive tumor with a smooth and glossy surface was observed in the right hepatic lobe, and the Glisson’s capsule remained intact. Moreover, in the patient’s left hepatic lobe, the first and second porta hepatis had been removed by the pressure, and the inferior vena cava was distorted.

**Figure 2:**
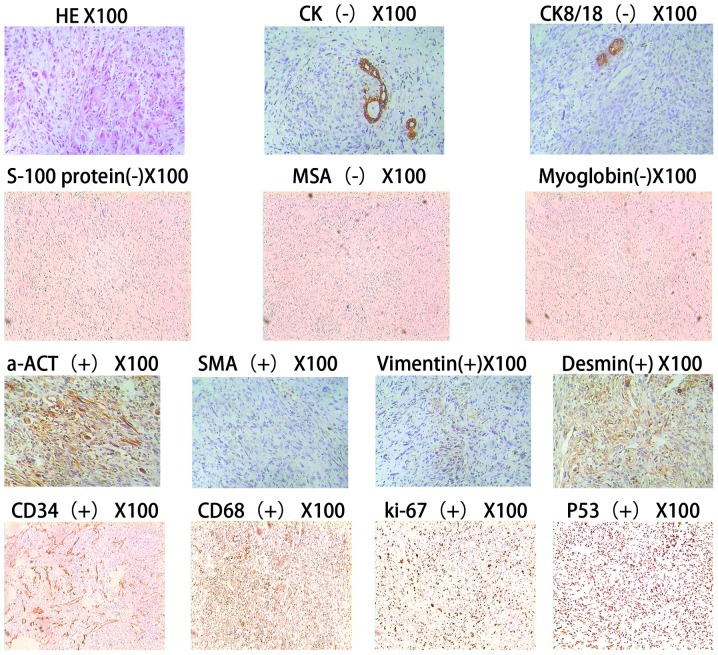
Light microscopy. The tumor cells exhibited embryonic mesenchymal differentiation and lacked epithelial characteristics. Moreover, the tumor cells demonstrated fusiform and star shapes, which have undetermined boundaries and clear heteromorphism. Changes in the interstitial mucus and the periodic acid-Schiff (PAS)-positive eosinophilic body, both inside and outside the tumor cells, were evident. The remnant hepatic cells and hyperplastic bile duct are visible in the boundaries of the tumor, and there were no changes in the hepatic cirrhosis. The immunohistochemistry results are as follows: Broad spectrum creatine kinase (CK)(−), cytokeratin 8/18(CK8/18)(−), S-100(−), MSA(−), myoglobin(−), SMA foci(+), a-ACT foci(+), desmin foci(+), vimentin foci(+), CD34(+), CD68(+), Ki-67(+) and P53(+).

The following clinical pathology results were observed by the naked eye. The size of the tumor in the hepatic right lobe was ∼17.0 cm, and the incisal surface was occupied by a gray-yellow/red/white swelling. Additionally, the texture of the swelling was impalpable and soft. Necrosis was observed and the swollen tissue demonstrated the partial stick-slip phenomenon. Further clinicopathological results were obtained using a light microscope. Under the light microscope (Figs. 1 and 2), the tumor cells demonstrated embryonic mesenchymal differentiation and lacked epithelial characteristics. Moreover, the tumor cells exhibited fusiform and star shapes with undetermined boundaries and clear heteromorphism. In addition, changes in the interstitial mucus and the periodic acid-Schiff (PAS)-positive eosinophilic body, both inside and outside the tumor cells, were identified. Furthermore, the remnant hepatic cells and the hyperplastic bile duct were observed in the boundaries of the tumor, and there were no changes in hepatic cirrhosis. The postoperative pathological diagnosis was undifferentiated sarcoma in the right hepatic lobe without tumor tissue in the gall bladder.

The immunohistochemistry results were as follows: Broad spectrum creatine kinase (CK)(−), cytokeratin 8/18(CK8/18)(−), S-100(−), MSA(−), myoglobin(−), SMA foci(+), a-ACT foci(+), desmin foci(+), vimentin foci(+), CD34(+), CD68(+), Ki-67(+) and P53(+).

The karyotype analysis revealed that the chromosomal aberrations accounted for the remaining 5% of all chromosomes, which involved chromosomes 6, 11, 12 and 14, as well as the X chromosome. The aberrations included fourcentric chromosomes, deletion, disruption and marker chromosomes.

## Discussion

UESL is a rare hepatic mesenchymal tumor that was first reported and classified by Stocker *et al* in 1978 (1). The incidence of the disease has no significant gender difference. In addition, 90% of patients are children aged 6–10 years, and the diesease accounts for 5–8% of hepatic tumors in children. The tumor is mainly localized or found in the hepatic right lobe (59%), while it rarely develops in the hepatic left lobe (22%) or the bilateral lobe (20%). UESL typically has a diameter of 10–25 cm with a solitary clear boundary (6). Hemorrhaging, necrosis and cystic degeneration are frequently observed, while clinical manifestations include abdominal mass, pain, fever and, rarely, jaundice.

An ultrasonography scan reveals a mixed echoic mass containing an irregular anechoic region or multiple small capsular spaces of different sizes. The solid region exhibits a mixture of high- and low-level echos. By contrast, a computed tomography (CT) scan reveals cystic lesions with low density that is reflected as a fluid. Therefore, the difference between CT and ultrasonography results is a critical characteristic of this disease. However, both scans reveal cystic lesions when hemorrhage, necrosis and liquefaction account for the major part of the tumor. An enhanced CT scan of the tumor solid part, septation and pseudo-capsule shows different degrees of reinforcement. However, in the present study, MRI revealed that the T1W1 scan mainly detected a low or equal signal and high signal foci, which were a reflection of a hemorrhage in the tumor. Additionally, the T2W1 scan frequently exhibited a mixed high signal. Positron emission tomography (PET)-CT scans are also used in the diagnosis of UESL, and they have a critical diagnostic value for UESL patients, particularly those with metastasis of an extra-hepatic organ.

The immunohistochemistry results reveal the positive expression of SMA, a-ACT, desmin, vimentin and actin in UESL patients, and a minority of cases are positive for PCNA, CK8/18 and p53; while AFP, S-100, CEA, CA 19-9 and cytokeratin have negative expression. The indices of the patient were as follows: SMA foci(+), a-ACT foci(+), desmin foci(+), vimentin foci(+), broad spectrum CK(−), CK8/18(−), AFP(−), CEA(−) and CA19-9(−).

Generally, a definite diagnosis of UESL is not able to be determined preoperatively; the diagnosis relies on postoperative pathological analysis and immunohistochemical results. UESL ought to be differentiated from hepatoblastoma, embryonal rhabdomyosarcoma, hepatic mesenchymal hamartoma and hepatic echinococcosis. Hepaoblastoma, which mainly occurs in infants aged <3 years, is composed of primary hepatic parenchymal cells with small cellular heteromorphism, little karyokinesis, increased blood sinus and positive expression of endosomal membrane protein, vimentin and AFP revealed by immunohistochemistry. By contrast, embryonal rhabdomyosarcoma mainly occurs in infants aged <6 years, and the tumor is mainly composed of striated muscle maternal cells in different phases and primary mesenchymal cells. The myosin often exhibits strong positive expression by immunohistochemistry, and transverse striation may be observed by preservative associated transient hyperfluorescence (PATH) staining. With hepatic mesenchymal hamartoma, which mainly occurs in infants aged <1 year, the tumor is composed of a mucus matrix and a large number of star and fusiform primary mesenchymal cells, whereas the tumor cells themselves do not exhibit heteromorphism. By contrast, hepatic echinococcosis is a local parasitic disease that mainly occurs in adults aged 20–40 years. Casoni and complement fixation tests are used to diagnose this disease, and the positive rate is 90–95%, which has a critical diagnostic value. A case history combined with imaging analysis has been demonstrated to be beneficial for the discrimination of UESL. A previous study has revealed that UESL is often misdiagnosed as echinococcosis of the liver (2). Previous studies have demostrated that cases of UESL have exhibited an amplification and deletion in chromosomes 1q, 5p, 8p and 12q, and a translocation of 19q13.4 (7), as well as mutation of the p53 gene (8). Therefore, the detection of molecular genetics is beneficial for the differential diagnosis of UESL. In this case, chromosomal aberration accounted for the remaining 5% of all chromosomes, which invovled chromosomes 6, 11, 12 and 14, as well as the X chromsome. However, the patient’s parents’ and sisters’ chromosomes were normal, which indicated that genetic mutations may induce tumorigenesis, and thus further studies ought to be performed.

UESL is generally considered to be a highly invasive malignant tumor in hepatic primary mesenchymal tissue with distant metastasis. Lee *et al* described a case of UESL in a child with distant metastases in the lung and adrenal glands (4). The prognosis of UESL was not observed to correlate with the size and degree of differentiation of the tumor; however, it was found to correlate with invasion, diffusion and metastasis (9). In recent years, survival rates have significantly improved due to improvements in therapy, and long-term survival cases have been reported (9). The key management of the disease is total resection followed by postoperative combined therapeutic measures, including chemotherapy, radiotherapy and interventional therapy, which are able to significantly improve survival rates. Yu *et al* suggested that the best method for improving survival is total resection, regardless of whether the tumor is ruptured (10). Uchiyama *et al* proposed that surgical resection combined with chemotherapy is the most effective therapy for UESL patients with tumor rupture. Additionally, the authors suggested that chemotherapy containing 3–4 types of drugs, such as adriamycin, cisplatin and others, demonstrated beneficial therapeutic effects and significantly improved survival rates (5). Li *et al* demonstrated that a UESL patient who underwent interventional therapy and surgical resection exhibited a prolonged survival compared with patients who underwent surgical resection only (3). McCarthy *et al* described a case of a pregnant UESL patient who underwent trans-catheter ablation to control the development of the tumor (11). Certain studies have demonstrated that hepatic transplantation is an effective therapeutic method for UESL patients whose tumor is not able to be resected or who have postoperative recurrence of the tumor. This method may potentially significantly improve survival rates and times (12).

In the present case, blockage of the blood flow in half the liver was performed while the patient was under total anesthesia on December 29, 2009. A massive tumor with a smooth and glossy surface was observed in the right hepatic lobe and the Glisson’s capsule remained intact. Moreover, in the patient’s left hepatic lobe, the first and second porta hepatis had been removed by the pressure, and the inferior vena cava had been distorted. The surgery ran smoothly. The hepatic right lobe and partial left internal lobe were resected. The patient recovered successfully, and a chemotherapy regimen of epirubicin at 20 mg for days 1–2 and cisplatin at 15 mg for days 1–3 was administered at 14-day intervals following surgery. Subsequently, epirubicin at 20 mg for days 1–2 and cisplatin at 15 mg for days 1–3 were administered in the second and third cycle of chemotherapy on July 27, 2010. Currently, the patient’s physical status is normal, and no local recurrence or metastasis has been observed. We conclude that preoperative and/or postoperative interventional therapy, combined with radiotherapy and chemotherapy, may improve survival rates and times in certain cases. The precise timing of the total surgical resection is crucial to prevent invasive growth of the tumor, and a liver transplantation is the most effective therapy for a patient whose tumor cannot be surgically resected.

## Figures and Tables

**Figure 3:**
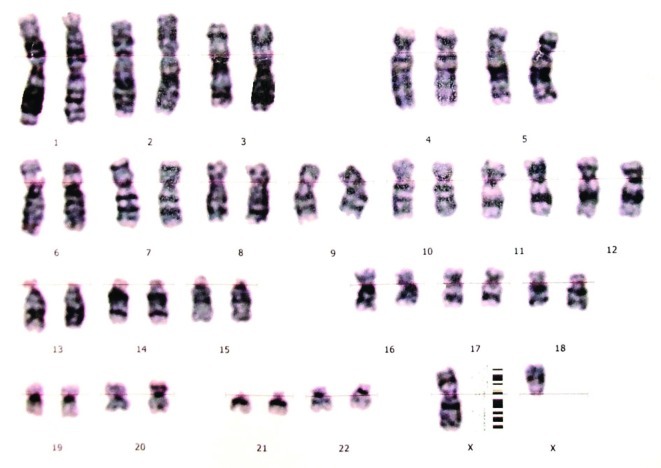
Karyotyping revealed a normal karyotype of 46 autosomes, and the XX chromosome accounts for 95% of all chromosomes. The chromosomal aberrations account for the remaining 5%, which involves chromosomes 6, 11, 12 and 14, as well as the X chromosome. Aberrations include fourcentric chromosomes, deletion, disruption and marker chromosomes, for example, the part deletion of the X karyotype.
